# Target screening and optimization of candidate compounds for breast cancer treatment using bioinformatics and computational chemistry approaches

**DOI:** 10.3389/fphar.2025.1467504

**Published:** 2025-05-09

**Authors:** Jian Xu, Xue Li, Yiduo Jia

**Affiliations:** ^1^ Shaoxing People’s Hospital, Shaoxing, China; ^2^ School of Medicine and Pharmacy, Wuhan University of Bioengineering, Wuhan, China; ^3^ School of Chemical Engineering and Pharmacy, Wuhan Institute of Technology, Wuhan, China; ^4^ Hubei Key Laboratory of Natural Medicinal Chemistry and Resource Evaluation, School of Pharmacy, Huazhong University of Science and Technology, Wuhan, China

**Keywords:** cancer, dock, targeted therapy, computational analysis, breast

## Abstract

**Objectives:**

This study aimed to identify critical therapeutic targets and design potent antitumor compounds for breast cancer treatment through an integrated bioinformatics and computational chemistry approach.

**Methods:**

We conducted initial screening and target intersection analysis to identify potential protein targets, highlighting the adenosine A1 receptor as a key candidate. Molecular docking and molecular dynamics (MD) simulations were performed to evaluate the binding stability between selected compounds and the human adenosine A1 receptor-Gi2 protein complex (PDB ID: 7LD3). A pharmacophore model was constructed based on binding information to guide the virtual screening of additional compounds with activity. Furthermore, we designed and synthesized a novel molecule based on this model, followed by *in vitro* biological evaluation using MCF-7 breast cancer cells.

**Results:**

Compound 5 exhibited stable binding to the adenosine A1 receptor, as confirmed by docking and MD simulations. Pharmacophore-based screening identified compounds 6–9 with strong binding affinities. These findings guided Molecule 10, which was rationally designed and synthesized, showing potent antitumor activity against MCF-7 cells with an IC50 value of 0.032 µM, significantly outperforming the positive control 5-FU (IC50 = 0.45 µM).

**Conclusion:**

This study advances the understanding of molecular interactions in breast cancer therapy and demonstrates the potential of Molecule 10 as a highly effective therapeutic candidate. Integrating reverse drug screening, molecular modelling, and *in vitro* validation provides a robust platform for future drug discovery in breast cancer treatment.

## 1 Introduction

Breast cancer, a malignant tumour originating in breast tissue, remains one of the most prevalent cancers worldwide, primarily affecting women but also occurring in men at lower frequencies. Its incidence and mortality rates vary significantly across regions due to genetic, environmental, hormonal, and lifestyle factors (Neagu et al., 2023). Extensive research has sought to unravel the complexities of breast cancer biology, focusing on its aetiology, risk factors, and molecular mechanisms underlying its initiation, progression, and metastasis. These studies have laid the foundation for developing personalized and targeted therapeutic strategies (Herdiana et al., 2023).

**SCHEME 1:**
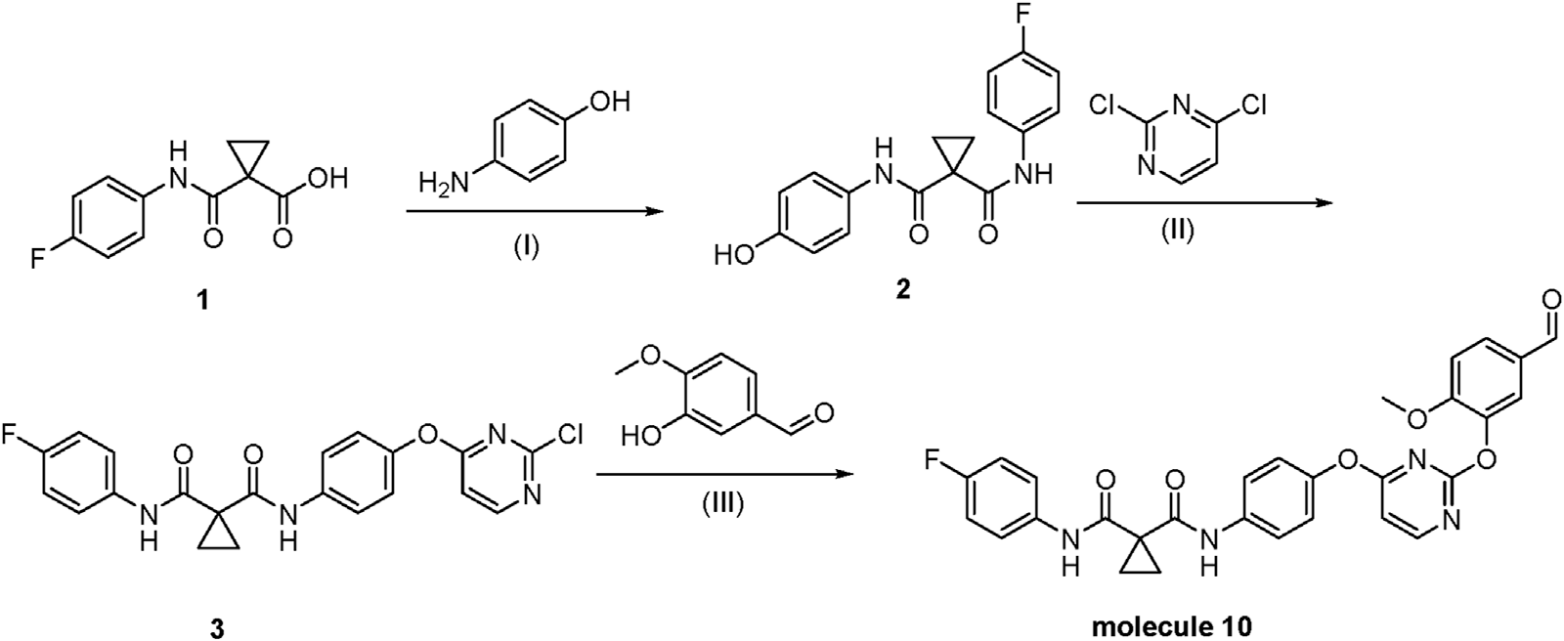
Regends and conditions.

Cell models are critical in advancing breast cancer research (Zhang et al., 2024). Among these, the estrogen receptor-positive (ER+) MCF-7 cell line, derived from human breast cancer tissue, is widely used to investigate the estrogen dependency of breast cancer and to evaluate therapies targeting estrogen signalling pathways. In contrast, the MDA-MB cell line, characterized by its lack of estrogen receptor expression (ER-), is primarily employed for studying more aggressive breast cancer behaviours, such as metastasis and drug resistance. Together, these cell models provide valuable insights into the heterogeneity of breast cancer and facilitate the development of novel treatment strategies (Özgen et al., 2025).

Target-based drug discovery has emerged as a promising approach for accelerating drug development in cancer research (Tauro et al., 2025). By leveraging bioinformatics and computational chemistry, researchers can efficiently identify key molecular targets and screen potential compounds with high binding affinities. For instance, Sunitinib (Azimian and Dastmalchi, 2023), an angiogenesis inhibitor used in the treatment of renal cell carcinoma and gastrointestinal stromal tumours, was developed through the application of computational chemistry methods to optimize its molecular structure, enhancing its selectivity and activity against specific tyrosine kinase targets. Similarly, Pazopanib, employed for treating renal cell carcinoma and soft tissue sarcoma, was identified using computational techniques focused on its interactions with multiple receptor tyrosine kinases, ultimately leading to its clinical approval (Menezes et al., 2021). This strategy enhances our understanding of molecular interactions in cancer and supports the rational design of structure-based therapies (Gahbauer et al., 2023).

This study employs a bioinformatics and computational chemistry approach to identify novel therapeutic targets and compounds for breast cancer treatment, providing a scientifically rational framework for drug discovery. By integrating computational tools such as molecular docking, molecular dynamics simulations, and pharmacophore modelling, we aim to enhance the precision and efficiency of identifying compounds that specifically interact with key proteins involved in breast cancer progression. This method contributes to a deeper understanding of the molecular interactions in breast cancer therapy and aligns with the growing need for personalized and targeted treatment strategies. Our approach offers a promising pathway for discovering potential lead compounds, ultimately advancing the development of more effective and tailored therapies for breast cancer patients.

## 2 Materials and methods

The experimental procedure was performed with an Intel Xeon CPU E5-2650, 2.00 GHz processor, using a Windows 10 operating system and a 4 GB NVIDIA Quadro 2000 graphics card. VMD 1.9.3 was used as a 3D visualization window.

**FIGURE 1:**
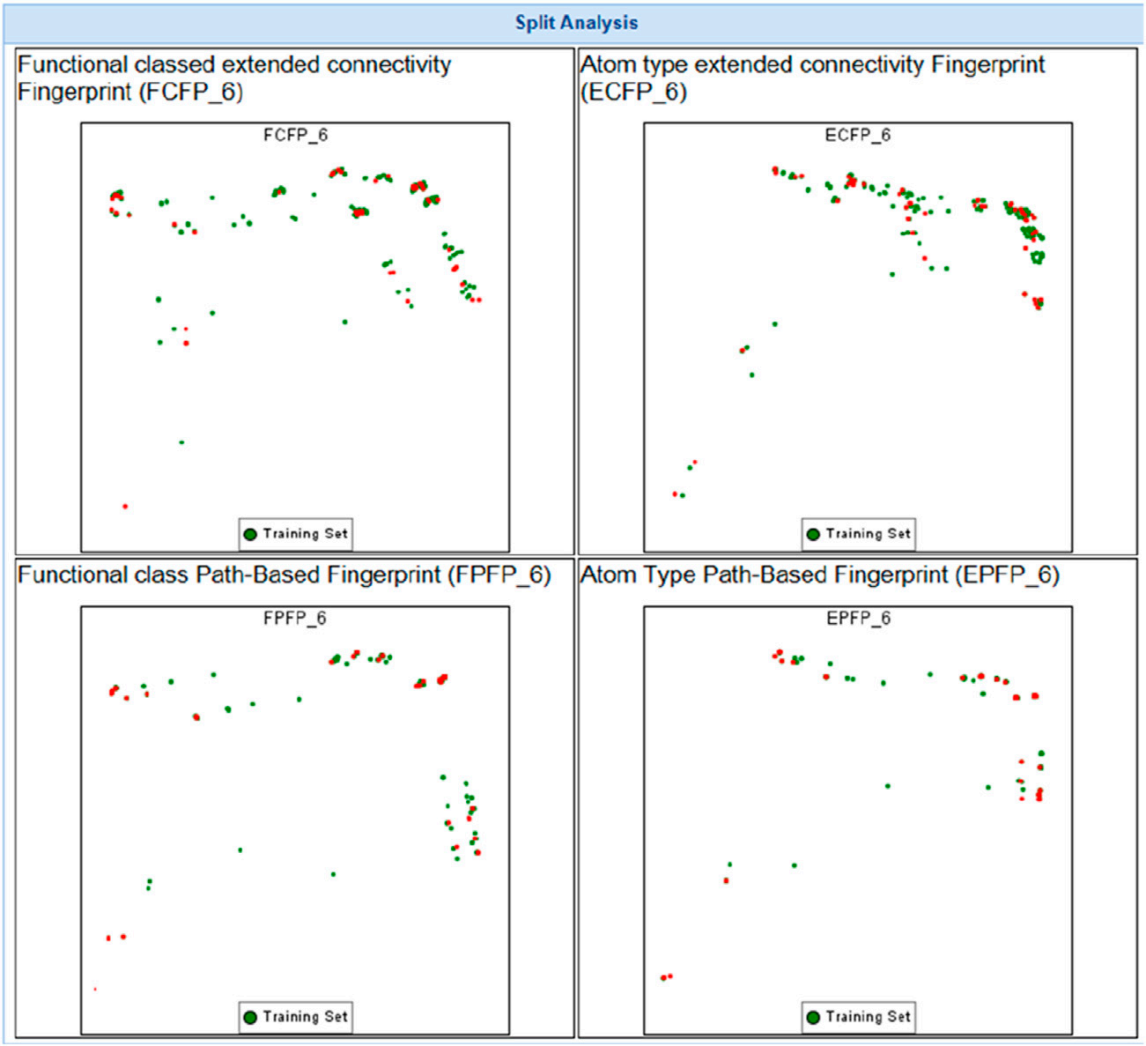
Split analysis of 249 compounds.

This study systematically analyzed 23 compounds selected from 23 publications (Adams et al., 2002; Benedekovic et al., 2020; Benedeković et al., 2020; Bheeram et al., 2019; Bohyun et al., 2016; Devendar et al., 2014; Dulong et al., 2013; Gerhard et al., 2010; Haiwen et al., 2007; Holger et al., 2012; Huzaifa et al., 2018; Ihmaid et al., 2022; Jojart et al., 2021; Khan et al., 2022; Maria et al., 2012; Nakhjiri et al., 2012; Nejat et al., 2018b; Noor et al., 2021; Rebeka et al., 2020; Satish Kumar et al., 2019; Tamizharasan et al., 2020; Thomas et al., 2005; Wukun et al., 2012; Wukun et al., 2011), each demonstrating significant inhibitory effects on MDA-MB and MCF-7 breast cancer cell lines. Initially, three-dimensional quantitative structure-activity relationship (3D-QSAR) analyses were performed to evaluate the spatial diversity of these compounds. Through conformational optimization, a total of 249 distinct conformers were generated. Subsequently, a split analysis, as depicted in Figure 1, was conducted to construct five pharmacophore models. These models, defined based on spatial differences, were screening tools to identify key structural features influencing biological activity. Each of the five pharmacophores exhibited significant spatial diversity. From these, the most potent compound within each pharmacophore category was selected, and detailed information about these compounds is presented in Table 1.

**TABLE 1:** Compounds of anti-MCF-7 and MDA-MB tumour cells.

Compounds	Structural formula	IC50 (µM)	
		MCF-7	MDA-MB
1 (Khan et al., 2022)	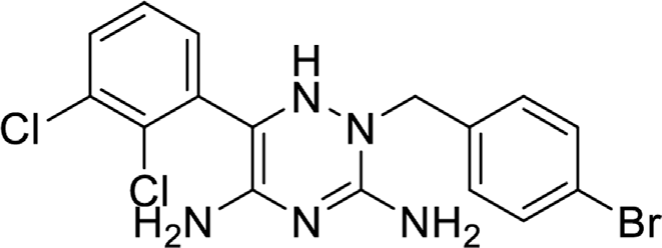	3.4	4.7
2 (Benedeković et al., 2020)	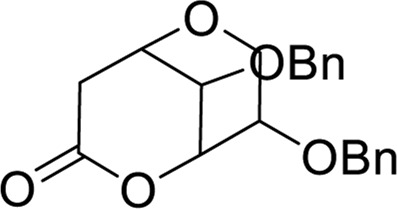	0.21	0.16
3 (Nejat et al., 2018a)	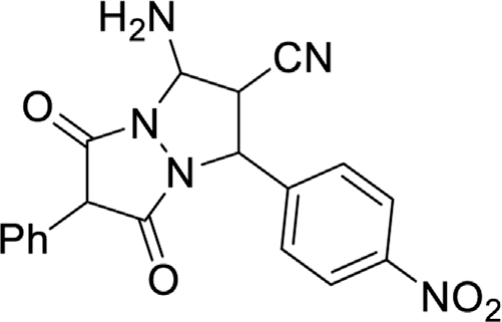	3	2.5
4 (Tamizharasan et al., 2020)	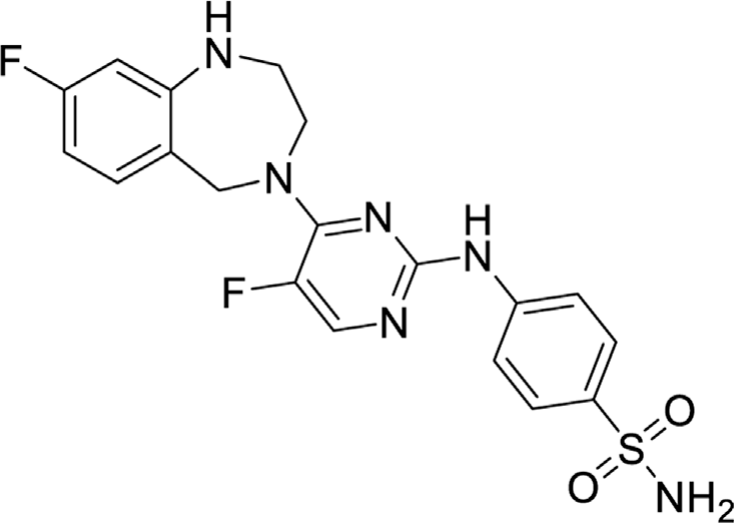	0.57	0.42
5 (Ihmaid et al., 2022)	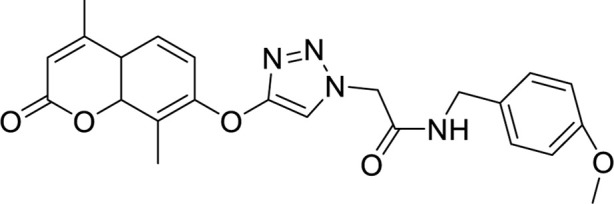	3.47	1.43

The compounds listed in Table 1 exhibit significant structural diversity, which may lead to interactions with multiple targets within biological systems, thereby increasing the complexity of drug action and providing a broader range of potential targets. The SwissTargetPrediction Database (http://swisstargetprediction.ch) was employed to predict these potential interactions. Using the chemical structures of the selected compounds as input and specifying “*Homo sapiens*” as the species, potential therapeutic targets were identified, offering valuable insights for further research.

### 2.1 Virtual screening

#### 2.1.1 Intersection of compounds against breast cancer

Five anticancer drugs, designed with distinct philosophies, share common antitumor targets against MDA-MB and MCF-7 breast cancer cell lines. The references for these five drugs, as introduced in Table 1, provide detailed descriptions of their spatial configurations. Utilizing the online tool “https://bioinfogp.cnb.csic.es/tools/venny/index.html,” we conducted an intersection analysis of the 500 predicted targets of these five compounds, revealing a shared target (Sihombing et al., 2024).

#### 2.1.2 Screening of compounds against breast cancer through the PubChem database

To further screen the targets and study the interactions between the targets, we screened the targets of two proteins through the PubChem Database (https://pubchem.ncbi.nlm.nih.gov/) (Lv et al., 2023), and the source used was the keywords “MDA-MB and MCF-7” were used to screen out the protein targets of the drug.

#### 2.1.3 Molecular docking simulation and validation

In this process, we created a ligand library using Discovery Studio 2019 Client, performed docking with CHARMM to refine ligand shapes and charge distribution, and analyzed binding interactions between compounds and drug targets. Table 4 contains the LibDockScore results, representing the best-scoring outcomes based on the binding sites reported in the existing literature. We selected the best poses based on LibDock scores, filtering targets with scores over 130, providing valuable insights into the binding mechanisms for our research (Guo et al., 2024).

#### 2.1.4 The stability of the docked complexes studied by molecular dynamics (MD) simulation

Molecular dynamics (MD) simulations using GROMACS 2020.3 analyzed protein-ligand binding dynamics. Protein structures were optimized with the AMBER99SB-ILDN force field (Vieira et al., 2023), and water molecules were modelled with the TIP3P model (Dorrani and Mohebbi, 2023). ACPYPE calculated ligand charges and generated GAFF force field-compatible files (Zaremba et al., 2023). Simulations employed cubic boxes with a minimum atom-box boundary distance of 0.8 nm, hydrated with SOL water at 1000 g/L density. Chloride ions replaced solvent water for electrical neutrality. An initial energy minimization step relaxed the system, followed by a 150 ps restrained Molecular dynamics (MD) simulation at 298.15 K. Unrestricted Molecular dynamics (MD) simulations with a time step of 0.002 ps were performed for 15 ns, maintaining isothermal-isobaric conditions at 298.15 K and 1 bar pressure, controlled by thermostats and barostats.

#### 2.1.5 The distribution of dynamic binding positions of compound 5

We extensively summarized the motion trajectory of the molecule interacting with the target using VMD 1.9.3 software. This comprehensive analysis spans from the initial to the 8220th frame, with data recorded every 200 frames. This frequency of data capture allowed for meticulous observation and documentation of the molecule’s dynamics throughout this timeframe, facilitating a thorough understanding of the molecular binding process to the target (Hsieh et al., 2023). Employing this systematic analytical approach aids in elucidating the molecule’s dynamic behaviour during the binding process, potential intermediate states, and the temporal sequence of binding events.

#### 2.1.6 Binding force analysis

We utilized Discovery Studio 2019 Client to analyze the interaction between molecules and proteins (Baroroh et al., 2023). From a quantum chemical perspective, the interaction between molecules and proteins involves three primary binding forces: hydrogen bonds, electrostatic forces, and hydrophobic forces. Hydrogen bonds, characterized by interactions between hydrogen atoms and electronegative atoms such as oxygen or nitrogen, play a crucial role in stabilizing the interaction between molecules and proteins. Electrostatic forces, arising from the attraction or repulsion between charged particles, facilitate the interaction between oppositely charged regions of proteins and molecules, enhancing their binding affinity. Additionally, hydrophobic forces, stemming from the tendency of nonpolar molecules to avoid water, drive the clustering of hydrophobic residues within proteins, thereby promoting interactions with hydrophobic regions of molecules and strengthening the overall binding stability. Collectively, these binding forces dictate the mode, strength, and functional implications of molecular-protein interactions, offering critical insights into protein structure-function relationships and guiding drug design strategies in scientific research.

#### 2.1.7 Protein subcellular localization

Subcellular localization refers to the precise cellular location of a protein or its gene expression product, encompassing compartments such as the nucleus, cytoplasm, and cell membrane. This spatial organization is crucial for the protein’s proper function, as it ensures access to the necessary chemical environment and interacting factors. Misplacement can disrupt cellular processes, making understanding protein localization essential for studying gene functions, protein interactions, and their mechanisms[20].

PSORT is a computer program used to predict the subcellular location of proteins based on their amino acid sequences and source information. To predict subcellular localization, the amino acid sequences of target proteins are obtained from the UniProt Database, and these sequences are then input into the PSORT II online software (https://psort.hgc.jp/). PSORT II provides predictions regarding the subcellular location of the target proteins (Li et al., 2023).

#### 2.1.8 Docking study

We have identified the interaction sites of drugs and their targets through subcellular localization, confirming where the compounds exert their effects on the human adenosine A1 receptor-Gi2 protein target. By reviewing relevant literature on this target, we found diverse structural designs for drugs targeting the A1 receptor. Despite significant differences in their molecular structures, these compounds exhibit strong interactions with the target. Therefore, we conducted a molecular docking study to evaluate the interactions between the target and these compounds. This approach allowed us to assess the binding affinities and interaction mechanisms, providing insights into the design principles for developing effective human adenosine A1 receptor-Gi2 protein inhibitors.

#### 2.1.9 Pharmacophores construction

The compounds exhibit significant differences in spatial structures, yet they all demonstrate strong interactions with the target. Based on their spatial structures and interactions with the target, we constructed a pharmacophore model using Discovery Studio 2019. The pharmacophore model was then used to screen our designed compound library (Jia et al., 2023).

The pharmacophore model represents the essential features responsible for the biological activity of the compounds. These features include hydrogen bond acceptors and donors, hydrophobic regions, and aromatic rings critical for the binding to the target. By leveraging this model, we were able to identify potential compounds from our library that fit the pharmacophore and are likely to interact effectively with the target. This approach not only streamlined the identification of promising compounds but also provided a rational basis for designing and optimizing new inhibitors targeting the human adenosine A1 receptor-Gi2 protein.

### 2.2 Chemistry

#### 2.2.1 Synthesis route of molecule 10

All reagents and starting materials were obtained from commercial suppliers and used without further purification. The progress of the reactions was monitored using analytical thin-layer chromatography (TLC) on GF254 precoated plates, visualized with a UV lamp and iodine (I2) staining. Column chromatography used Acme silica gel (200–300 mesh) with dichloromethane, methanol, petroleum ether, and ethyl acetate as eluents. ^1^H NMR spectra were recorded at 300 and 500 MHz, and ^13^C NMR spectra at 75 and 125 MHz, using a Bruker Avance spectrometer. Deuterated chloroform (CDCl3), dimethyl sulfoxide-d6 (DMSO-*d*_6_), or deuterium oxide (D2O) were used as solvents, with tetramethylsilane (TMS) as the internal standard. Chemical shifts are reported in parts per million (δ) downfield from TMS. NMR signal abbreviations include s = singlet, br s = broad singlet, d = doublet, t = triplet, q = quartet, m = multiplet, and dd = doublet of doublets. Melting points were measured using a Micro melting point apparatus SGWX-4 without correction. LCMS spectra were recorded using a QSTAR XL hybrid MS/MS system with methanol as the solvent. High-performance liquid chromatography (HPLC) was conducted using UltiMate 3000 equipment comprising a Variable Wavelength Detector, Autosampler, and pump to determine product purity. The column used was an Acclaim^®^ 120 C18 (5 μm, 120 Å, 4.6 × 250 mm). The flow rate was set to 1.00 mL/min, with an injection volume of 5.0 µL and detection at 254 nm for 30 min. The solvents were: A: water; B: acetonitrile; C: water with 0.5% (vol/vol) trifluoroacetic acid. An isocratic elution with 85% B was performed from 0 to 7 min. Detailed synthetic procedures and spectroscopic data are provided in the Supporting Information.

### 2.3 Activity

#### 2.3.1 *In vitro* activity assay

Due to the restricted availability of MDA-MB cell lines, we cannot evaluate the anti-breast cancer activity using MCF-7 cell lines. To inhibit MCF-7 cell lines, we chose the MTT ratio, cell calculation, and selective adsorption of the synthesized drugs.

Human liver tumour MCF-7 cell lines (kindly presented by Prof. Xiang Ming, Tongji Medical College, Huazhong University of Science and Technology); DMEM high sugar medium (Hyclone); penicillin, streptomycin (Hyclone, production lot no. 16677148); 0.25% trypsin, dimethyl sulfoxide (DMSO). Pentafluorouracil (5-FU) (analytical purity, Sigma, USA); fetal bovine serum (Zhejiang Tian hang Biotechnology Co., Ltd.); phosphate buffer solution (PBS) (Shanghai Double Helix Biotechnology Co., Ltd.); compounds 10.

## 3 Results

### 3.1 Virtual screening

#### 3.1.1 Screening of anti-breast cancer protein targets of five compounds

**FIGURE 2:**
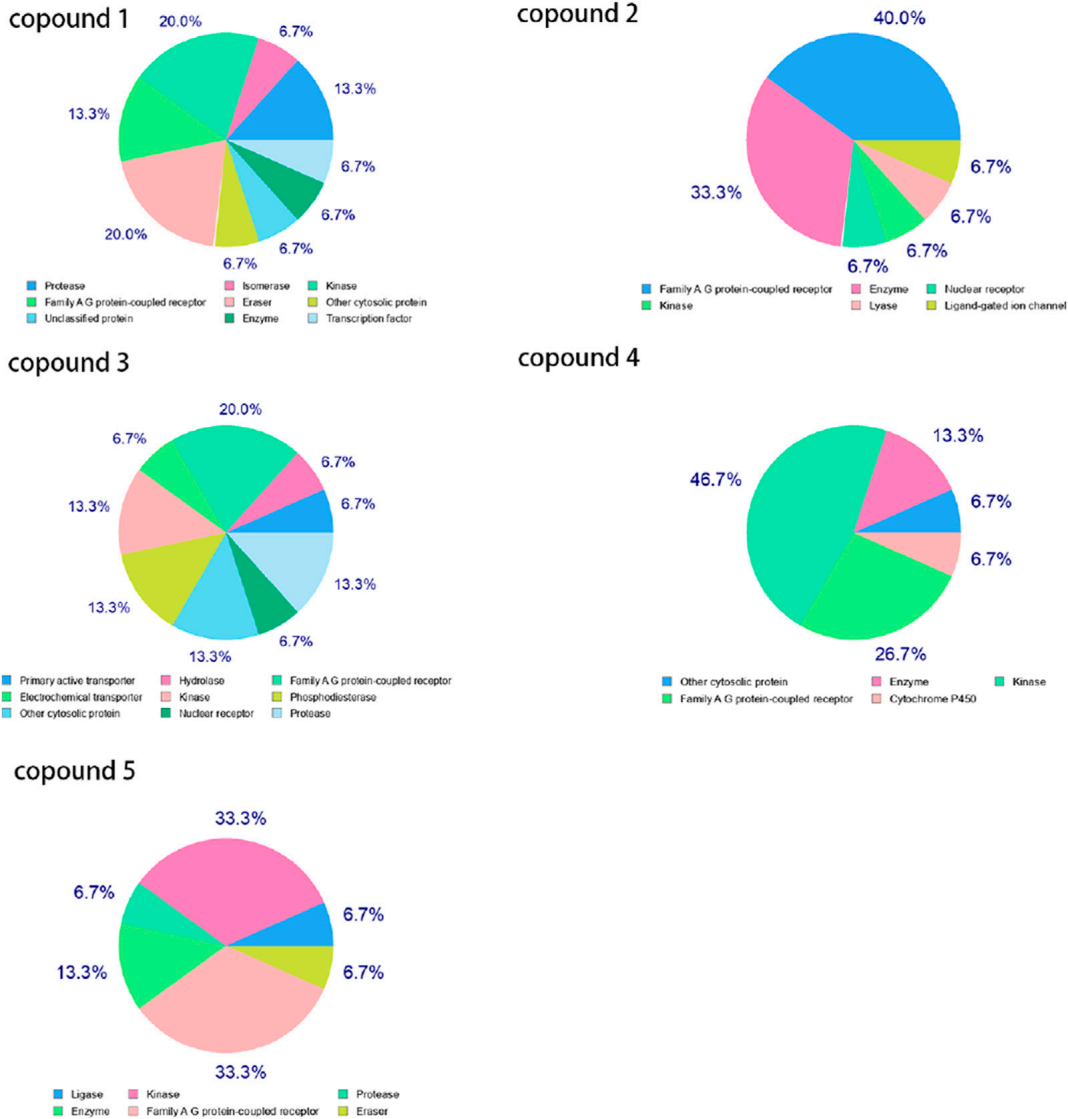
Target classes of five Compounds.

We screened 500 targets using the SwissTargetPrediction Database, and Figure 2 displays the targets for five compounds. The Family A G protein-coupled receptor ranks among the top three.

#### 3.1.2 Intersection of anti-breast cancer protein targets of five compounds

**FIGURE 3:**
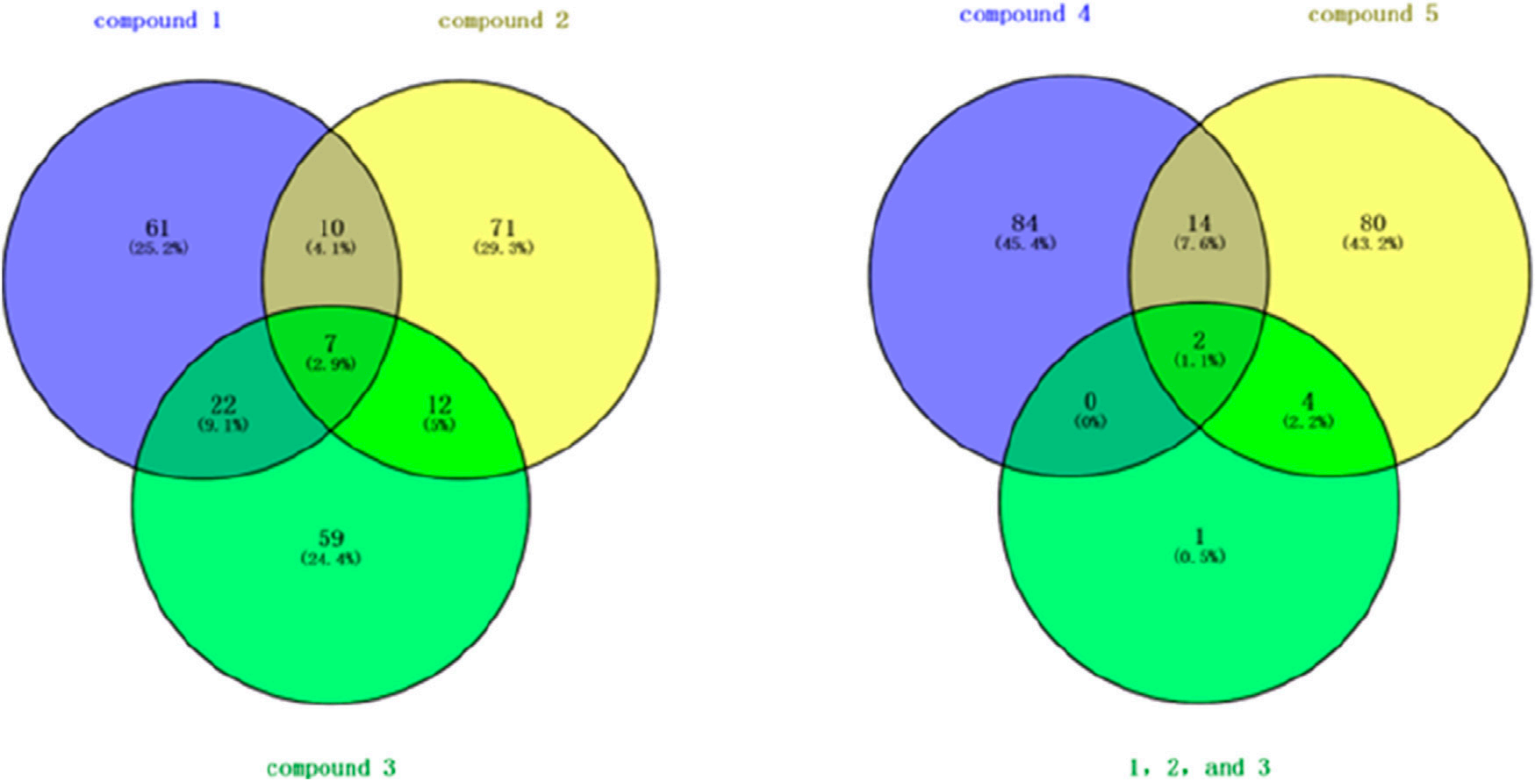
Venn diagram of targets from five compounds. 1. Note: “1, 2, and 3” represent the intersecting targets of Compound 1, Compound 2, and Compound 3 shown in the left diagram.

Through Venny 2.1.0 (csic.es)(https://bioinfogp.cnb.csic.es/tools/venny/index.html) online software, we screened two targets from 500 targets of three compounds, as shown in Figure 3. The targets are shown in Table 2.

**TABLE 2:** The screened targets by Venn.

Public protein	Target
Adenosine receptor A2a	ADORA2A
Adenosine receptor A1	THE ADENOSINE A1 RECEPTOR

#### 3.1.3 Screening of anti-breast cancer protein targets of five compounds through PubChem database

After screening proteins via PubChem (nih.gov) (https://puchem.ncbi.nlm.nih.gov/) in Table 3, we discovered the significant importance of THE ADENOSINE A1 RECEPTOR protein in anti-breast cancer therapy. THE ADENOSINE A1 RECEPTOR protein, a type of adenosine receptor, plays a crucial role in regulating biological processes such as cell proliferation, apoptosis, and migration. Studies have indicated a close correlation between the expression levels of THE ADENOSINE A1 RECEPTOR protein in breast cancer cells and tumour growth, metastasis, and treatment sensitivity. Its regulatory role in cellular signalling pathways makes it a potential target for anti-breast cancer therapy. Therefore, further research into THE ADENOSINE A1 RECEPTOR protein and exploring compounds' interaction may provide new therapeutic strategies and drug design directions for breast cancer treatment. Conversely, ADORA2A protein shows lower relevance to breast cancer proteins. Consequently, subsequent studies will focus on the screening and analysis of THE ADENOSINE A1 RECEPTOR protein, aiming to delve deeper into its potential role and clinical applications in breast cancer treatment.

**TABLE 3:** Results of screened targets by PubChem Database.

Target	PDB ID
Adenosine A1 receptor	5N2S,6D9H,7LD3
ADORA2A	6ZDR, 7PX4, 8C9W, 8FYN, 8GNE, 8RQQ, 6LPJ, 6WQA, 8CU7, 8JWY, 8PWN

#### 3.1.4 Molecular docking simulation and validation

The adenosine A1 receptor plays multiple roles in the tumour microenvironment, particularly in breast cancer and other tumours. THE ADENOSINE A1 RECEPTOR is closely associated with antitumor immunity, apoptosis, and the inhibition of tumour proliferation. Upon activation, THE ADENOSINE A1 RECEPTOR mediates its effects via G protein-coupled signaling pathways, inhibiting cancer cell proliferation and promoting apoptosis. In breast cancer cell lines such as MDA-MB and MCF-7, the role of THE ADENOSINE A1 RECEPTOR extends beyond direct effects on tumour cells to modulate the immune system. Specifically, ADENOSINE A1 RECEPTOR activation promotes T cell-mediated antitumor immunity, enhancing the body’s immune response against cancer cells.

In contrast, ADORA2A (Adenosine A2A receptor) primarily contributes to immune suppression in the tumour microenvironment. ADORA2A regulates immune cells, such as T and dendritic cells, to facilitate tumour immune evasion. This suppression of the immune response enables the tumour to escape immune detection and grow. Therefore, in breast cancer research, THE ADENOSINE A1 RECEPTOR is prioritized over ADORA2A due to its pivotal role in regulating immune responses, inhibiting tumour growth, and promoting apoptosis. While ADORA2A’s role in immune evasion and suppression is important, THE ADENOSINE A1 RECEPTOR holds more potential for directly combating tumour progression, making it an ideal target for cancer therapy.

We also conducted molecular docking studies using Discovery Studio 2019 Client to investigate the interactions between five compounds and three target proteins. In this study, we used LibDockScore to quantify the binding affinity between molecules. Compounds with a LibDockScore exceeding 130 during the docking process were considered significant binders, as shown in Table 4. This threshold was selected to prioritize compounds with higher binding affinity, thus identifying them as strong candidates for further investigation. Notably, none of the five compounds showed significant interactions with ADORA2A-related targets. Therefore, based on the current molecular docking results, the anticancer activity of these compounds appears to be unrelated to ADORA2A.

**TABLE 4:** Results of docking.

Target	Compound	libdockscore	Absolute energy	Relative energy
5N2S	1	110.463	60.3866	4.91131
	2	126.078	57.4398	19.6871
	3	116.618	57.4614	5.61654
	4	111.041	77.7491	0.0629889
	5	133.462	57.6704	7.47434
6D9H	1	80.3367	56.4111	0.0113962
	2	98.9731	55.9383	18.1855
	3	90.9335	57.6229	5.77804
	4	98.525	80.3409	0.189017
	5	103.311	60.1029	9.90685
7LD3	1	102.325	66.3654	9.57802
	2	116.588	39.6037	1.85097
	3	63.8847	56.3795	4.53461
	4	130.194	78.0161	0.33005
	5	148.673	53.5358	3.33969

#### 3.1.5 The stability of the docked complexes studied by molecular dynamics (MD) simulation

**FIGURE 4:**
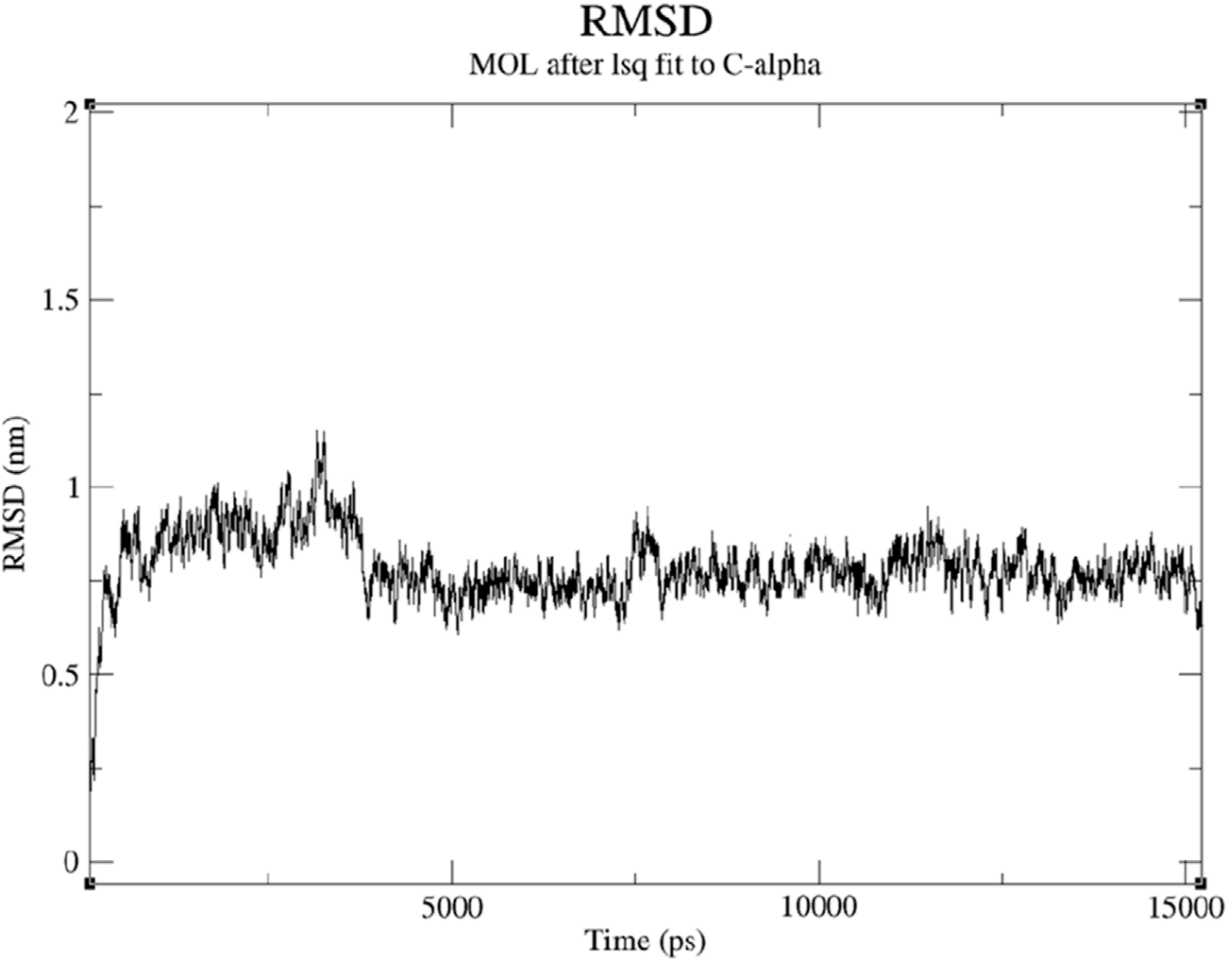
RMSD of Compound 5 with protein target (PDB ID: 7LD3) in 15000ps.

Root Mean Square Deviation (RMSD) is a standard metric used to assess the structural changes of molecules during molecular dynamics simulations, reflecting the deviation between the molecule’s conformation at each time step and its initial structure. This study’s RMSD values remained around 1 nm, indicating a relatively stable binding state between Compound 5 and the 7LD3 protein. The maintenance of RMSD values around 1 nm suggests that no significant conformational changes occurred in the molecule during the simulation. The RMSD values of compound 5 with the 7LD3 protein remained relatively stable, with a relative variation of around 0.5 ns in Figure 4. This relative stability in the binding state indicates an intense and persistent interaction.

Compound 5 and the 7LD3 protein are promising candidates for further drug design and development efforts.

A stable binding state is crucial for drug design as it ensures reliable interactions between the molecule and the target protein, potentially enhancing the drug’s efficacy and safety. Therefore, the analysis of RMSD values provides deeper insights into the interaction characteristics between Compound 5 and the 7LD3 protein, serving as important reference data for subsequent drug design and clinical research.

#### 3.1.6 The distribution of dynamic binding positions of molecules

**FIGURE 5:**
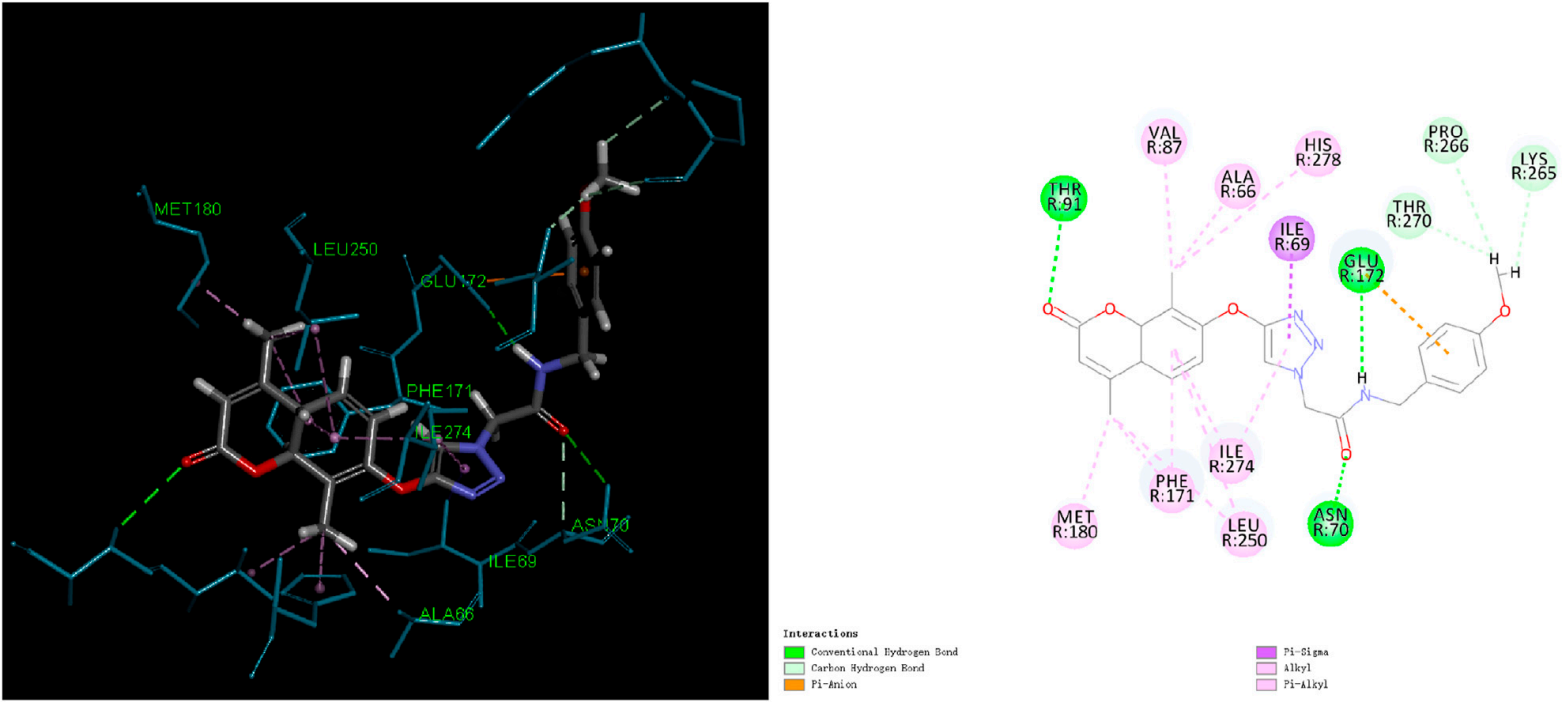
2D and 3D diagrams of Compound 5 with protein target (PDB ID: 7LD3).

Through the analysis of the binding frequency of molecules at different locations, preferred binding regions on the protein surface can be identified in Supplementary Figure S1. Certain regions may exhibit a higher binding affinity for molecules, indicating potential drug-binding sites. The duration of molecule residence at different locations can assess the stability of the interaction between the molecule and the protein target. Prolonged residence at specific locations suggests a stable binding, whereas short residence times correspond to weak, transient interactions. Within the 15000ps simulation timeframe, the molecule’s motion range, as illustrated in Figure 5, indicates a relatively stable state at the binding site on the protein. Based on this binding site, molecular docking between the molecule and the protein target was conducted.

#### 3.1.7 Binding force analysis

In light of the Molecular dynamics (MD) simulation results, we performed a binding force analysis using Discovery Studio’s receptor-ligand interaction calculation tool. In Figure 5, the interactions between protein (PDB ID: 7LD3) and Compound 5 mainly comprised hydrogen bonds, Electrostatic and Hydrophobic. For hydrogen bonds, residue 91, residue 70, and residue 172 formed three conventional hydrogen bonds with bond lengths of 2.0Å and 3.11Å. Residue 265, residue 266, and residue 270 formed three carbon-hydrogen bonds. For Electrostatic: residue 124 formed one Pi-Anion bond. For Hydrophobic, residues 66, 87, 274, 171, 278, 180, and 250 formed four Pi-Alkyl bonds, one Pi-Sigma bond, and four Alkyl bonds.

#### 3.1.8 Protein subcellular localization

In Table 5, the predominant localization of THE ADENOSINE A1 RECEPTOR within the cytoplasm (71.87%) underscores its potential involvement in cellular interactions within this compartment. Consequently, Compound 5 is implicated in interacting with the protein within the cytoplasmic milieu. This localization pattern suggests that THE ADENOSINE A1 RECEPTOR may play pivotal roles in cytoplasmic signalling cascades, cellular metabolism, or other essential processes within this dynamic cellular region. The high proportion of THE ADENOSINE A1 RECEPTOR in the cytoplasm implies its significance in orchestrating cellular responses to extracellular stimuli or modulating intracellular signalling pathways critical for cell homeostasis. Therefore, understanding the interactions between Compound 5 and THE ADENOSINE A1 RECEPTOR in the cytoplasmic context holds promise for elucidating their functional implications and potential therapeutic applications in cellular processes governed by THE ADENOSINE A1 RECEPTOR-mediated signalling.

**TABLE 5:** Protein subcellular localization results.

Drug	Protein	Location (k = 23)	UniProt ID	PDB ID
Compound 5	The adenosine A1 receptor	Extra: 71.87%	P30542	7LD3
		Plus 18.75%		
		Nucl: 6.25% also: 3.13%		

Compounds inhibit the activity of the human adenosine A1 receptor-Gi2 protein target and their molecular docking study. We identified compounds with significant inhibitory activity against the human adenosine A1 receptor-Gi2 protein through a comprehensive literature review. The research papers on these compounds have an impact factor greater than 10 (IF > 10).

Ultimately, we selected four compounds demonstrating notable inhibitory activity against this target, as summarized in Table 6. To further validate the inhibitory effects of these compounds, we employed molecular docking techniques, docking each of the four compounds with the 7LD3 target. The optimal docking conformations for each compound were identified and are detailed in Table 7.

**TABLE 6:** Structures against the human adenosine A1 receptor-Gi2-protein.

Compounds	Structural formula
6 (Draper-Joyce et al., 2021)	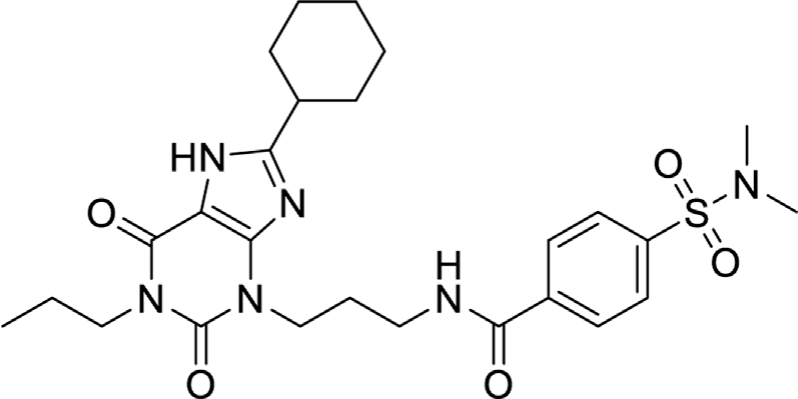
7 (Liu et al., 2019)	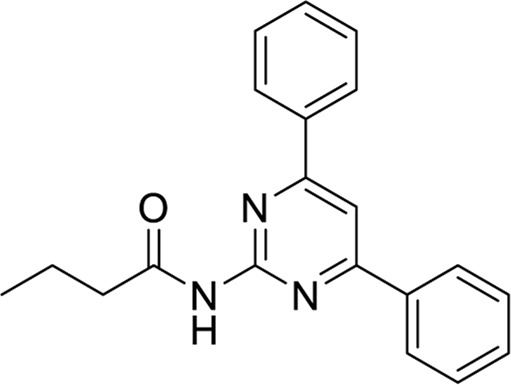
8 (Meibom et al., 2017)	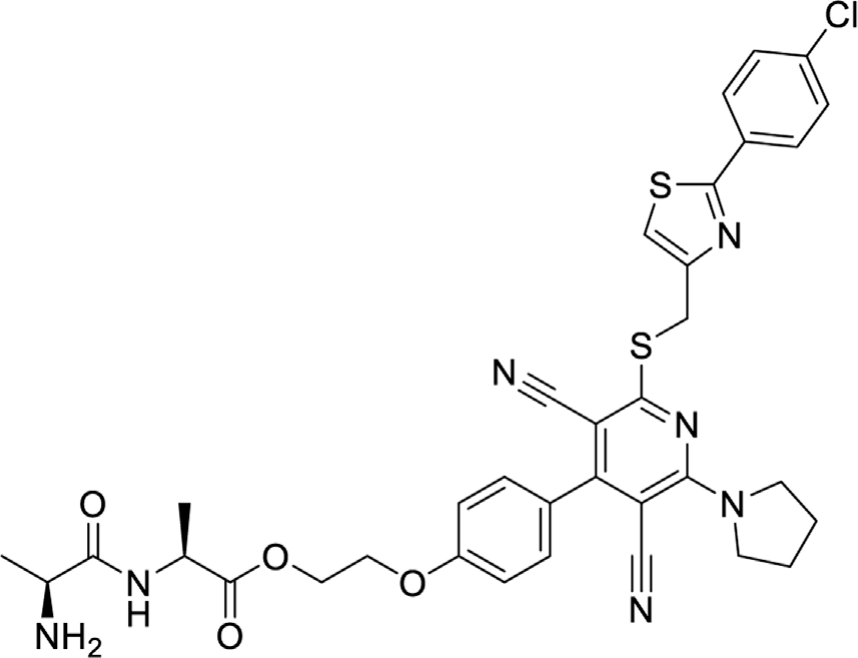
9 (Vu et al., 2004)	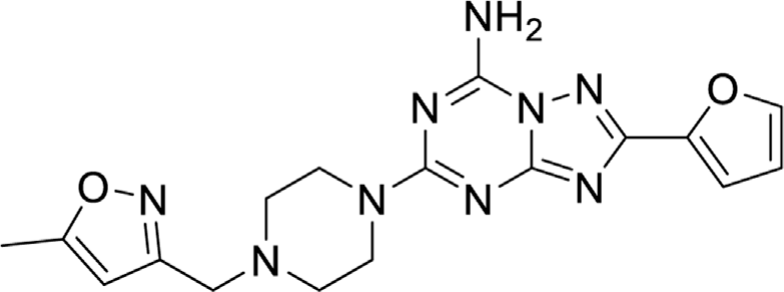

**TABLE 7:** Results of docking in protein target with compounds.

Target	compound	libdockscore	Absolute energy	Relative energy
7LD3	6	109.963	53.4481	5.32973
	7	114.504	78.0218	5.11853
	8	76.097	97.4955	0.801548
	9	136.729	81.5171	2.3624

#### 3.1.9 Pharmacophores construction

Leveraging insights into compound-target interactions, in Table 8, we systematically constructed 22 pharmacophore models. Subsequent scrutiny of our compound library involved meticulous assessment of fit values against these models. Notably, this process unearthed two pharmacophore models demonstrating robust compatibility with compound 10. Specifically, pharmacophore model Molecule7_03 exhibited a fit value of 3.07, while pharmacophore model Molecule7_04 displayed a fit value of 3.05, indicating their strong affinity for compound 10.

**TABLE 8:** Results of screening in pharmacophores with compounds.

Pharmacophores	Best docking pose	Fit value
Molecule7_01	Molecule9-2	1.30226
Molecule7_02	Molecule15-11	1.95561
Molecule7_03	Molecule10-4	3.07071
Molecule7_04	Molecule10-1	3.0506
Molecule7_05	Molecule13-12	2.47614
Molecule7_06	Molecule8-6	2.01747
Molecule8_01	0	0
Molecule8_02	0	0
Molecule8_03	Molecule12-8	2.09077
Molecule8_04	0	0
Molecule8_05	Molecule12-2	1.19133
Molecule8_06	Molecule3-10	1.05892
Molecule9_01	0	0
Molecule9_02	0	0
Molecule9_03	0	0
Molecule9_04	0	0
Molecule9_05	0	0
Molecule9_06	0	0
Molecule9_07	0	0
Molecule9_08	0	0
Molecule9_09	0	0
Molecule9_10	0	0

### 3.2 Chemistry

#### 3.2.1 Synthesis route of molecule 10

Compound 1 was transformed into compound two as (^1^H NMR is shown in Supplementary Figure S2) via amide condensation. Subsequently, compound 2 underwent nucleophilic substitution with 1,4-dichloropyrimidine to yield compound 3(^1^H NMR is shown in Supplementary Figure S3). Compound 3 was further subjected to nucleophilic substitution with 3-hydroxy-4-methoxybenzaldehyde to obtain the final product, molecule 10(^1^H NMR and ^13^C NMR, respectively are shown in Supplementary Figure S4 and Supplementary Figure S5, the LCMS of molecule 10 is shown in Supplementary Figure S6). For detailed synthetic procedures, refer to Supplementary 2.1 Chemistry.

### 3.3 Activity

#### 3.3.1 *In vitro* activity assay

We conducted an MTT assay to evaluate the biological activity of compound 10, using 5-fluorouracil (5-Fu) as a positive control. The experimental results demonstrated that compound 10 significantly inhibits breast cancer cells, as shown in Table 9. The IC50 value of compound 10 was determined to be 0.032 ± 0.026 µM (The database is shown in Supplementary Table S9), indicating a potent inhibitory activity at relatively low concentrations. These findings suggest that compound 10 possesses promising anti-breast cancer properties, providing a basis for further pharmacological studies and development. It is important to acknowledge that the biological activity of compound 10 was tested only on the MCF-7 breast cancer cell line. While these results are promising, they are limited to this particular cell type, which may not fully represent the heterogeneity of breast cancer. Future studies should include additional cell lines, such as MDA-MB cells, to assess the broader efficacy of compound 10 across different breast cancer subtypes and provide a more comprehensive evaluation of its potential therapeutic value. The detailed experimental procedures for the *in vitro* antitumor activity assay are provided in Supplementary 3.1.

**TABLE 9:** MCF-7 cell viability and IC50 analysis.

Compounds	IC50 (µM)
1 (Khan et al., 2022)	3.4
2 (Benedeković et al., 2020)	0.21
3 (Nejat et al., 2018a)	3
4 (Tamizharasan et al., 2020)	0.57
5 (Ihmaid et al., 2022)	3.47
Molecule 10	0.32
5-FU (Positive cotrol)	0.45

Note: 1. IC50 values were calculated using nonlinear regression analysis. 2. Positive control: 5-FU (5-Fluorouracil).

### 3.4 Role of the adenosine A1 receptor in cancer signaling pathways and its therapeutic potential

In the context of cancer, THE ADENOSINE A1 RECEPTOR’s signalling mechanisms contribute to the suppression of tumour growth and metastasis. It achieves this by modulating the tumour microenvironment, enhancing antitumor immune responses, and interfering with key processes such as angiogenesis and cellular survival. Activation of THE ADENOSINE A1 RECEPTOR has been linked to the inhibition of cancer cell growth and induction of apoptosis through various molecular pathways, including the suppression of NF-κB activity, a key driver of inflammation and tumorigenesis.

This study contributes significantly to understanding THE ADENOSINE A1 RECEPTOR’s therapeutic potential by identifying key binding sites and interactions through molecular docking and dynamics simulations. Our findings highlight that compound 10 interacts favourably with THE ADENOSINE A1 RECEPTOR, suggesting its potential as a targeted therapy for breast cancer. By exploring THE ADENOSINE A1 RECEPTOR’s involvement in cancer signalling pathways, this study not only validates its role in tumour suppression but also opens the door for further research into THE ADENOSINE A1 RECEPTOR-targeted therapeutic agents that could complement existing cancer treatments.

## 4 Conclusion

In conclusion, this study employed a comprehensive bioinformatics and computational chemistry approach to identify novel targets and active compounds for breast cancer treatment. Initial screening and intersection analyses revealed that THE ADENOSINE A1 RECEPTOR was a critical protein target. Molecular docking and molecular dynamics (MD) simulations demonstrated the stable binding of Compound 5 to the 7LD3 protein, suggesting its potential as a lead compound. Guided by a pharmacophore model constructed from the binding interactions of active compounds (6–9), we designed and synthesized Molecule 10, which exhibited significant antitumor activity against MCF-7 cells, with an IC50 value of 0.032 µM, underscoring its potential as a promising therapeutic candidate.

Furthermore, 500 target proteins were initially screened using the SwissTargetPrediction Database, and Family A G protein-coupled receptors were found to rank among the top three targets. An intersection analysis using Venny 2.1.0 online software identified two common targets for three compounds, providing further insights into their shared potential in breast cancer therapy. Protein screening through the PubChem Database highlighted the significant importance of the ADENOSINE A1 RECEPTOR protein, with lower relevance for ADORA2A.

We analyzed the interaction characteristics between the compounds and proteins, identifying five compounds with significant interactions. Given the substantial spatial differences among these compounds, we constructed 22 pharmacophore models and screened a custom-designed compound library, identifying molecules with strong potential for further development.

In future studies, Compound 10 will be tested in animal models to evaluate its vivo efficacy and potential therapeutic benefits in breast cancer treatment. Additionally, efforts will be made to optimize its pharmacokinetic properties, including bioavailability and metabolic stability, to enhance its clinical potential. This study primarily relied on *in vitro* assays and computational predictions, which may not fully capture the complexity of biological systems. The exclusive use of MCF-7 cells and computational models may limit the generalizability of the findings. Therefore, further studies incorporating additional *in vitro* models and *in vivo* validation are essential to confirm the therapeutic potential of the identified compounds.

(Ⅰ) HOBT, EDCI, DMF, r.t., 3.5 h; (Ⅱ) K_2_CO_3_, DMF, 80°C, 6 h; (Ⅲ) Cs_2_CO_3_, DMF, 100°C. Through a three-step synthesis, we obtained molecule 10, which demonstrated promising antitumor activity against MCF-7 cells in MTT assays, with an IC50 value of 0.032 µM. This study provides important theoretical support and candidate compounds for designing and developing therapeutics in breast cancer treatment.

## Data Availability

The datasets presented in this study can be found in online repositories. The names of the repository/repositories and accession number(s) can be found in the article/supplementary material.

## References

[B1] Adams M. Louise J. J. Rosemary A. Pringle J. H. Bell S. C. (2002). Changes in tenascin-C isoform expression in invasive and preinvasive breast disease. Cancer Res. 62, 3289–3297. 12036947

[B2] Azimian F. Dastmalchi S. (2023). Recent advances in structural modification strategies for lead optimization of tyrosine kinase inhibitors to explore novel anticancer agents. Curr. Med. Chem. 30, 2734–2761. 10.2174/0929867329666220920092908 36125833

[B3] Baroroh U. Biotek M. Muscifa Z. S. Destiarani W. Rohmatullah F. G. Yusuf M. (2023). Molecular interaction analysis and visualization of protein-ligand docking using Biovia Discovery Studio Visualizer, 2, 22–30.

[B4] Benedeković G. Popsavin M. Kovačević I. Kojić V. Rodić M. Popsavin V. (2020). Synthesis, antiproliferative activity and SAR analysis of (-)-cleistenolide and analogues, Eur. J. Med. Chem., 202 112597, 10.1016/j.ejmech.2020.112597 32653698

[B5] Benedekovic G. Popsavin M. Kovacevic I. Kojic V. Rodic M. Popsavin V. (2020). Synthesis, antiproliferative activity and SAR analysis of (-)-cleistenolide and analogues. Eur. J. Med. Chem. 202, 112597. 10.1016/j.ejmech.2020.112597 32653698

[B6] Bheeram V. R. Malla R. R. Kumari S. Saha A. Mukkamala S. B. (2019). Cytotoxic Effect of Photoluminescent RE3+ doped Ca3(PO4)2 nanorods on breast cancer cell lines. IRBM 40, 270–278. 10.1016/j.irbm.2019.05.001

[B7] Bohyun M. Yong Joo P. Gi Ho S. Yunmi L. Ki Hyun K. (2016). Synthesis and antitumor activity of (−)-bassianolide in MDA-MB 231 breast cancer cells through cell cycle arrest. Bioorg. Chem. 69, 64–70. 10.1016/j.bioorg.2016.09.008 27676608

[B8] Devendar P. Singh S. Srinivas K. V. N. S. Luqman S. Sarfaraz A. Zehra A. (2014). Synthesis of cyclic 1,9-acetal derivatives of forskolin and their bioactivity evaluation. Eur. J. Med. Chem. 87, 735–744. 10.1016/j.ejmech.2014.10.013 25305717

[B9] Dorrani H. Mohebbi A. J. J. o.E. T. (2023). A comparative study of TIP4P-2005, SPC/E, SPC, and TIP3P-Ew models for predicting water transport coefficients using EMD and NEMD simulations, 32, 138–161.

[B10] Draper-Joyce C. J. Bhola R. Wang J. Bhattarai A. Nguyen A. T. Cowie-Kent I. (2021). Positive allosteric mechanisms of adenosine A1 receptor-mediated analgesia. Nature 597, 571–576. 10.1038/s41586-021-03897-2 34497422 PMC8711093

[B11] Dulong C. Fang Y. J. Gest C. Zhou M. H. Patte-Mensah C. Mensah-Nyagan A. G. (2013). The small GTPase RhoA regulates the expression and function of the sodium channel Nav1.5 in breast cancer cells. Int. J. Oncol. 44, 539–547. 5 in breast cancer cells. 10.3892/ijo.2013.2214 24337141

[B12] Gahbauer S. Correy G. J. Schuller M. Ferla M. P. Doruk Y. U. Rachman M. (2023). Iterative computational design and crystallographic screening identifies potent inhibitors targeting the Nsp3 macrodomain of SARS-CoV-2, 120. e2212931120. 10.1073/pnas.2212931120 PMC9926234 36598939

[B13] Gerhard R. Kerstin B. Anja W. Brigitte K. Silke B. Ingo O. (2010). Synthesis and biological activities of transition metal complexes based on acetylsalicylic acid as neo-anticancer agents. J. Med. Chem. 53, 6889–6898. 10.1021/jm101019j 20857911

[B14] Guo X. Wang L. Zhang J. Liu Q. Wang B. Liu D. (2024). Thwarting resistance: MgrA inhibition with methylophiopogonanone a unveils a new battlefront against S. aureus, 10 15. 10.1038/s41522-024-00485-w PMC10899606 38413623

[B15] Haiwen Z. Solomon V. R. Changkun H. Gerardo U. Hoyun L. (2007). Synthesis and *in vitro* cytotoxicity evaluation of 4-aminoquinoline derivatives. Biomed. & Pharmacother. 10.1016/j.biopha.2007.04.007 PMC7125724 17555912

[B16] Herdiana Y. Sriwidodo S. Sofian F. F. Wilar G. Diantini A. J. M. (2023). Nanoparticle-based antioxidants in stress signaling and programmed cell death in breast cancer treatment, 28, 5305. 10.3390/molecules28145305 PMC10384004 37513179

[B17] Holger B. Sara K. Ulrike S.-B. Claudia C. Alexandra S. Stefanie A. (2012). Modulation of CXCR3 ligand secretion by prostaglandin E2 and cyclooxygenase inhibitors in human breast cancer. Breast Cancer Res. 14, R30. 10.1186/bcr3115 22333315 PMC3496148

[B18] Hsieh I.-M. Lin B. Mahbub H. Carter Z. Jebur M. Cao Y. (2023). Field demonstration of intensified membrane distillation for treating oilfield produced waters from unconventional wells. Desalination 564, 116771. 10.1016/j.desal.2023.116771

[B19] Huzaifa U. Doga K. Nahit R. (2018). Biosynthesis of zinc oxide nanoparticles using Albizia lebbeck stem bark, and evaluation of its antimicrobial, antioxidant, and cytotoxic activities on human breast cancer cell lines. Int. J. Nanomedicine Vol. 14, 87–100. 10.2147/ijn.s186888 PMC6304255 30587987

[B20] Ihmaid S. K. Aljuhani A. Alsehli M. Rezki N. Alawi A. Aldhafiri A. J. (2022). Discovery of triaromatic flexible agents bearing 1, 2, 3-Triazole with selective and potent anti-breast cancer activity and CDK9 inhibition supported by molecular dynamics, 1249.131568.

[B47] Jia Y. Li S. Zhang Y. Qi M. (2023). Design and synthesis of cyclopropanedicarboxamide-based hybrid drugs. Contemporary Chemical Research, 179–181.

[B21] Jojart R. Tahaei S. A. S. Trungel-Nagy P. Kele Z. Minorics R. Paragi G. (2021). Synthesis and evaluation of anticancer activities of 2- or 4-substituted 3-(N-benzyltriazolylmethyl)-13α-oestrone derivatives. J. Enzyme Inhib. Med. Chem. 36, 58–67. 10.1080/14756366.2020.1838500 33121276 PMC7598997

[B22] Khan M. K. Siddiqui H. Sharif R. Guzel M. Wahab A.-t. Yousuf S. (2022). Lamotrigine derivatives‐synthesis, anti‐cancer, and anti‐MDR‐bacterial activities, 1264.133277.

[B23] Li J. Zou Q. Yuan L. J. M. T.-N. A. (2023). A review from biological mapping to computation-based subcellular localization. Mol. Ther. Nucleic Acids 32, 507–521. 10.1016/j.omtn.2023.04.015 37215152 PMC10192651

[B24] Liu X. Liu J. Wu J. Huang G. Liang R. Chung L. W. (2019). Asymmetric total synthesis of cerorubenic acid-III. J. Am. Chem. Soc. 141, 2872–2877. 10.1021/jacs.8b12647 30721058

[B25] Lv Y. Mou Y. Su J. Liu S. Ding X. Yuan Y. (2023). The inhibitory effect and mechanism of Resina Draconis on the proliferation of MCF-7 breast cancer cells: a network pharmacology-based analysis, 13, 3816. 10.1038/s41598-023-30585-0 PMC9992681 36882618

[B26] Maria P. Wukun L. Adelheid H. Ulrich A. Ronald G. (2012). Synthesis, characterization and *in vitro* antitumour activity of a series of novel platinum(II) complexes bearing Schiff base ligands. Eur. J. Med. Chem. 53, 168–175. 10.1016/j.ejmech.2012.03.053 22534185

[B27] Meibom D. Albrecht‐Küpper B. Diedrichs N. Hübsch W. Kast R. Krämer T. (2017). Neladenoson bialanate hydrochloride: a prodrug of a partial adenosine A1 receptor agonist for the chronic treatment of heart diseases. 12 728–737. 10.1002/cmdc.201700151 28488817

[B28] Menezes T. M. da Silva Neto A. M. Gubert P. Neves J. L. J. J. o.M. L. (2021). Effects of human serum albumin glycation on the interaction with the tyrosine kinase inhibitor pazopanib unveiled by multi-spectroscopic and bioinformatic tools, 340.116843.

[B29] Nakhjiri M. Safavi M. Alipour E. Emami S. Atash A. F. Jafari-Zavareh M. (2012). Asymmetrical 2,6-bis(benzylidene)cyclohexanones: synthesis, cytotoxic activity and QSAR study. Eur. J. Med. Chem. 50, 113–123. 10.1016/j.ejmech.2012.01.045 22341788

[B30] Neagu A.-N. Whitham D. Bruno P. Morrissiey H. Darie C. A. Darie C. C. J. M. (2023). Omics-based investigations of breast cancer, 28, 4768. 10.3390/molecules28124768 PMC10302907 37375323

[B31] Nejat R. Mahjoub M. A. Hekmatian Z. Javidi M. A. Babashah S. (2018a). Zeolite-catalyzed synthesis of pyrazolo [1, 2-a][1, 2, 4] triazole-1, 3-dione derivatives as anti-breast cancer agents, 15, 1133–1143.

[B32] Nejat R. Mahjoub M. A. Hekmatian Z. Javidi M. A. Babashah S. (2018b). Zeolite-catalyzed synthesis of pyrazolo[1,2-a] [1,2,4]triazole-1,3-dione derivatives as anti-breast cancer agents. J. Iran. Chem. Soc. 15, 1133–1143. 10.1007/s13738-018-1310-6

[B33] Noor N. S. Kaus N. H. M. Szewczuk M. R. Hamid S. B. S. (2021). Formulation, characterization and cytotoxicity effects of novel thymoquinone-PLGA-PF68 nanoparticles. Int. J. Mol. Sci. 22, 9420. 10.3390/ijms22179420 34502328 PMC8431343

[B34] Özgen Ö. Çetin İ. Atalar F. Topçul M. R. J. C. Biology M. (2025). Alpha-mangostin and nab-paclitaxel in breast cancer cell models: improved antitumor efficacy through combination therapy. Cell. Mol. Biol. 71, 52–59. 10.14715/cmb/2025.70.1.6 39910941

[B35] Rebeka J. Seyyed Ashkan Senobar T. Péter T.-N. Zoltán K. Renáta M. Gábor P. (2020). Synthesis and evaluation of anticancer activities of 2- or 4-substituted 3-(N-benzyltriazolylmethyl)-13α-oestrone derivatives. J. Enzyme inhibition Med. Chem. 36, 58–67. 10.1080/14756366.2020.1838500 PMC7598997 33121276

[B36] Satish Kumar V. Rajkiran Reddy B. Sudip M. Purushotham U. Subbaiah G. Gurava Reddy A. V. (2019). Novel biosynthesized gold nanoparticles as anti-cancer agents against breast cancer: synthesis, biological evaluation, molecular modelling studies. Biomater. Adv. 10.1016/j.msec.2019.01.123 30889716

[B37] Sihombing I. N. N. Arsianti A. J. J. o.P. Research P. (2024). Network pharmacology prediction and molecular docking analysis on the mechanism of eugenol as a candidate against estrogen receptor-positive breast cancer. 12 837–851.

[B38] Tamizharasan N. Gajendran C. Kristam R. Sulochana S. P. Sivanandhan D. Mullangi R. (2020). Discovery and optimization of novel phenyldiazepine and pyridodiazepine based Aurora kinase inhibitors, Bioorg. Chem., 99 103800, 10.1016/j.bioorg.2020.103800 32283344

[B39] Tauro S. Dhokchawle B. Nahar D. Nadar S. Thakor E. Mohite P. J. D. D. S. (2025). Target-based vs phenotypic drug discovery: opportunities and challenges with evidence-based application, 25–45.

[B40] Thomas W. Ingo O. Brigitte K. Petra S. Daniela S. Thierry L. (2005). Synthesis and pharmacological evaluation of 1H-imidazoles as ligands for the estrogen receptor and cytotoxic inhibitors of the cyclooxygenase. J. Med. Chem. 48, 6516–6521. 10.1021/jm050190u 16190777

[B41] Vieira I. H. P. Botelho E. B. de Souza Gomes T. J. Kist R. Caceres R. A. Zanchi F. (2023). Visual dynamics: a WEB application for molecular dynamics simulation using GROMACS, BMC Bioinforma., 24 107, 10.1186/s12859-023-05234-y PMC10031864 36949402

[B42] Vu C. B. Peng B. Kumaravel G. Smits G. Jin X. Phadke D. (2004). Piperazine derivatives of [1, 2, 4] triazolo [1, 5-a] [1, 3, 5] triazine as potent and selective adenosine A2a receptor antagonists. J. Med. Chem. 47, 4291–4299. 10.1021/jm0498405 15294001

[B43] Wukun L. Kerstin B. Maria P. Adelheid H. Ulrich A. Ronald G. (2012). Synthesis, characterization, and *in vitro* studies of bis[1,3-diethyl-4,5-diarylimidazol-2-ylidene]gold(I/III) complexes. J. Med. Chem. 55, 3713–3724. 10.1021/jm3000196 22424185

[B44] Wukun L. Kerstin B. Maria P. Ulrich A. Adelheid H. Ronald G. (2011). NHC gold halide complexes derived from 4,5-diarylimidazoles: synthesis, structural analysis, and pharmacological investigations as potential antitumor agents. J. Med. Chem. 54, 8605–8615. 10.1021/jm201156x 22091836

[B45] Zaremba A. A. Zaremba P. Y. Zahorodnia S. D. J. S. R. (2023). *In silico* study of HASDI (high-affinity selective DNA intercalator) as a new agent capable of highly selective recognition of the DNA sequence. DNA Seq. 13, 5395. 10.1038/s41598-023-32595-4 PMC10070485 37012345

[B46] Zhang J. Liu J. Yue Y. Wang L. He Q. Xu S. (2024). The immunotoxin targeting PRLR increases tamoxifen sensitivity and enhances the efficacy of chemotherapy in breast cancer, 43, 173. 10.1186/s13046-024-03099-4 PMC11188579 38898487

