# Supplementary material for: Target screening and optimization of candidate compounds for breast cancer treatment using bioinformatics and computational chemistry approaches
Source: Front Pharmacol. 2025 May 9;16:1467504. doi: 10.3389/fphar.2025.1467504 (PMC12098522; doi:10.3389/fphar.2025.1467504)
Supplement: Supplementary file 1 [file DataSheet1.pdf]

Article

Target Screening and Optimization of Candidate Compounds for Breast Cancer Treatment Using Bioinformatics and Computational Chemistry Approaches

Jian Xu [1], Xue Li [2], Hong Zhu[4], Yiduo Jia [3*, 4]

[1] Shaoxing People's Hospital

[2] Wuhan University of Bioengineering

[3] Hubei Key Laboratory of Natural Medicinal Chemistry and Resource Evaluation, School of Pharmacy, Huazhong University of Science and Technology, Wuhan, 430030, China

[4] School of Chemical Engineering and Pharmacy, Wuhan Institute of Technology, Wuhan, 430000, China;

[3*, 4] Correspondence: jiayiduo0402@163.com

1. Experimental 3
1.1 Chemistry 4
1.1.1 Synthetic procedures and spectroscopic data 4
Figures: 6
Spectra (^{1}H NMR, ^{13}C NMR, and MS/HRMS) of molecule 10 7
1.2 Vitro anti-tumor activity experiment 8

1. Experimental

1.1 The distribution of dynamic binding positions of molecules

Through the analysis of the binding frequency of molecules at different locations, preferred binding regions on the protein surface can be identified. Certain regions may exhibit a hi gher binding affinity for molecules, indicating potential drug-binding sites. The duration of molecule residence at different locations can assess the stability of the interaction between the molecule and the protein target. Prolonged residence at specific locations suggests a st able binding, whereas short residence times correspond to weak, transient interactions. Wit hin the 15ns simulation timeframe, the molecule's motion range, as illustrated in Figure S 1, indicates a relatively stable state at the binding site on the protein. Based on this bindi ng site, molecular docking between the molecule and the protein target was conducted.

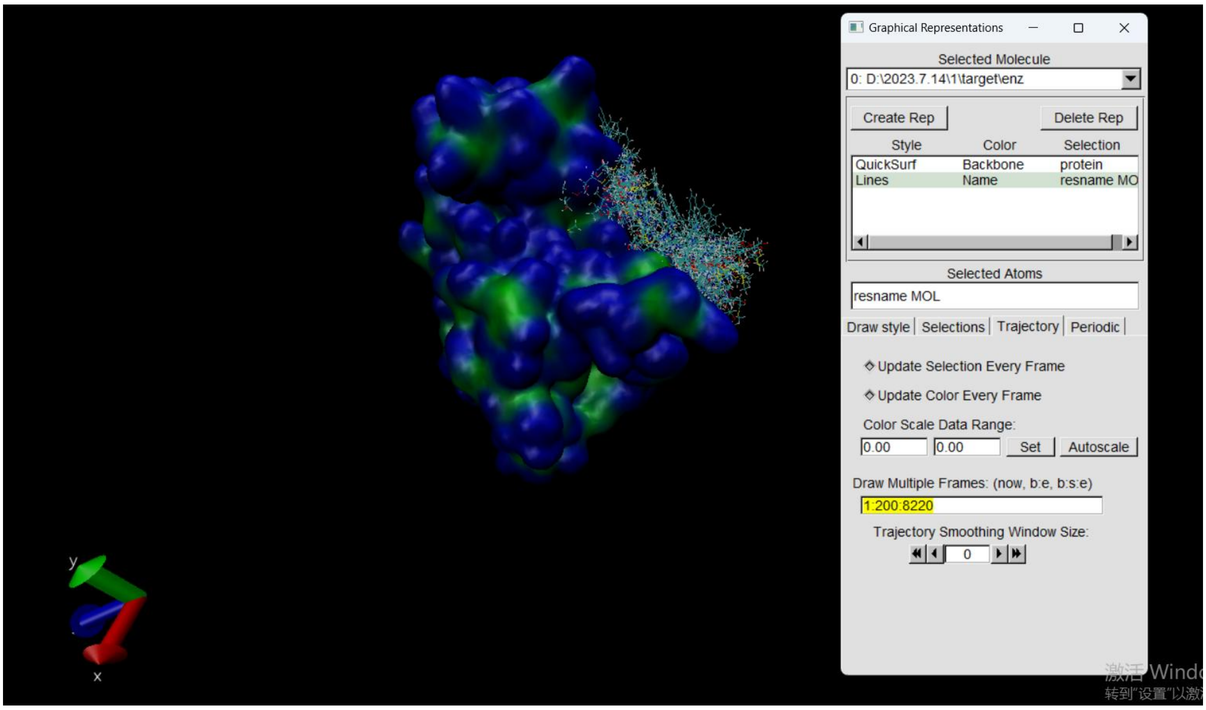

S1. The distribution of dynamic binding positions of molecules in 15ns.

2.1 Chemistry

2.1.1 Synthetic procedures and spectroscopic data

2.1.1.1 N-(4-fluorophenyl)-N-(4-hydroxyphenyl) cyclopropane-1,1-dicarboxamide. (compound **2**)

To a solution of Intermediate 3 (0.500 g, 2.240 mmol) in DMF, add HOBT (0.310 g, 2.300 mmol), EDC.HCl (0.520 g, 2.730 mmol) and then stir for 20 min at room temperature before adding 4-aminophenol (0.300 g, 2.730 mmol). The reaction was monitored by TLC for 3 h. After the reaction was completed, the product was obtained using alkali dissolution and acid precipitation, filtered and dried to obtain yellow solid. yield, 85.0%, ^{1}H NMR (400 MHz, DMSO-d6) δ 7.62 (d, J = 4.0 Hz, 1H), 7.15 (d, J = 9.2 Hz, 1H), 1.41 (s, 3H).

2.1.1.2 N-(4-((2-chloropyrimidin-4-yl)oxy)phenyl)-N-(4-fluorophenyl)cyclopropane -1,1-dicarboxamide (compound **3**)

Weigh compound 6 0.148 g (1 mmol) and intermediate 5 0.314 g (1 mmol) and dissolve in 8.00 ml of DMF, stir to dissolve and add anhydrous potassium carbonate 0.276 g (2.000 mmol), react at 80 ℃ for 6 h. After complete consumption of the reaction material by TLC, stop heating, leave the reaction solution to cool to room temperature, filter out the insoluble material and add the The reaction solution was slowly added dropwise to ice-cold distilled water to obtain a milk-like solution. A few drops of 1.000 mol/L hydrochloric acid were added slowly and gradually a precipitate was formed, stirred for 30 min and filtered, the filter cake was washed with distilled water, dried and slurried with petroleum ether to give a white solid. Yield, 75.6%. 1H NMR (400 MHz, DMSO-d6) δ 10.16 (s, 1H), 10.05 (s, 1H), 8.63 – 8.54 (m, 1H), 7.68 – 7.60 (m, 4H), 7.24 – 7.09 (m, 5H), 1.47 (s, 4H).

2.1.1.3 N-(4-fluorophenyl)-N-(4-((2-((5-formyl-2-methoxyphenoxy)methyl)pyrimidin-4-yl)oxy)phenyl)cyclopropane-1,1-dicarboxamide (**molecule 10**)

A mixture of Intermediate 7 (0.426 g, 1.000 mmol) Intermediate 8h (0.152 g, 1.000 mmol) and anhydrous potassium carbonate (0.277 g, 2.000 mmol) in 8.00 ml DMF, react overnight at 80 ℃, and stop after TLC monitoring to the end of the reaction. Heat, cool to room temperature, and filter. The filtrate was added dropwise to 100.00 ml ice water, a nd 1.000 mol/L dilute hydrochloric acid was slowly added dropwise until precipitation beg an to appear. A white precipitate gradually precipitated under stirring, filtered, and the filt er cake was washed with distilled water until it was neutral and dried. The obtained solid was separated by column chromatography, and the eluent was petroleum ether-ethyl acetate 2:1 to obtain yellow solid. yield, 77.7%. 1H NMR (400 MHz, DMSO-d6) δ 10.14 (s, 1 H), 10.06 (d, J = 4.0 Hz, 1H), 9.96 (s, 1H), 8.43 (dd, J = 16.0, 5.6 Hz, 1H), 7.70 – 7.5 3 (m, 7H), 7.39 (d, J = 8.0 Hz, 1H), 7.14 (ddd, J = 7.2, 5.6, 2.8 Hz, 4H), 6.79 (d, J = 5.6 Hz, 1H), 3.78 (s, 3H), 1.47 (d, J = 3.2 Hz, 4H), 1.37 – 1.13 (m, 2H). 13C NMR (1 01 MHz, DMSO-d6) δ 192.40, 171.62, 168.6, 164.26, 161.39, 152.30, 147.81, 146.48, 137. 06, 135.62, 135.11, 124.24, 123.83, 122.93, 122.00, 115.60, 115.38, 112.54, 103.57, 56.41, 31.87, 15.96. HPLC purity: 99.02%, retention time = 3.480 min. LC-MS(m/z): 543.0[M+H] +.

Figures:

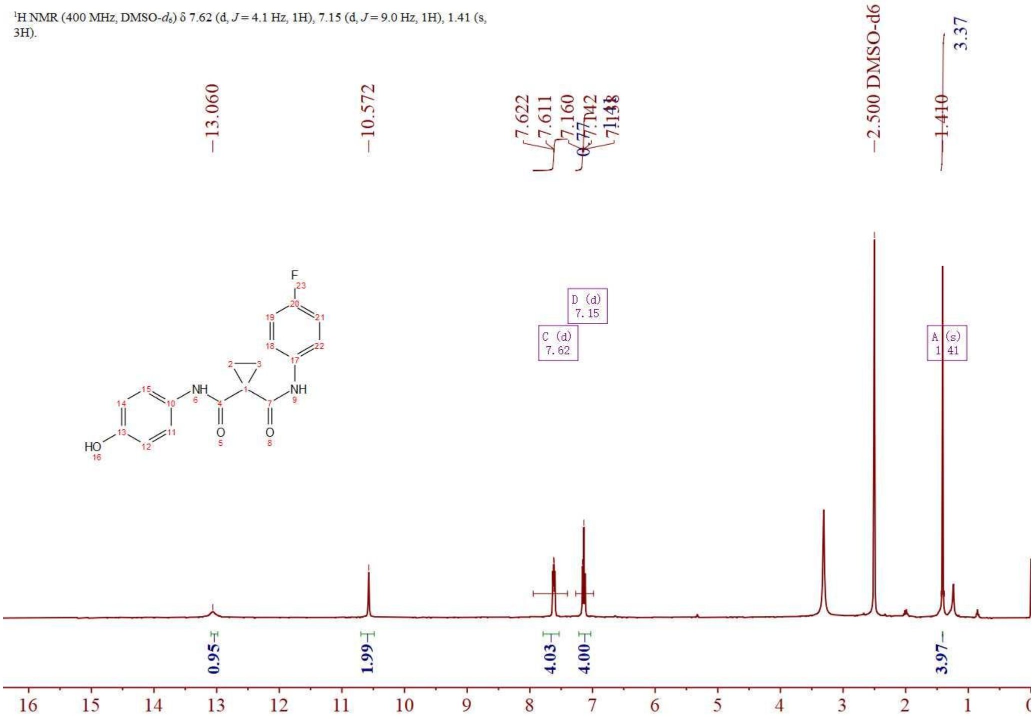

Figure S2. ¹H NMR of compound **2**

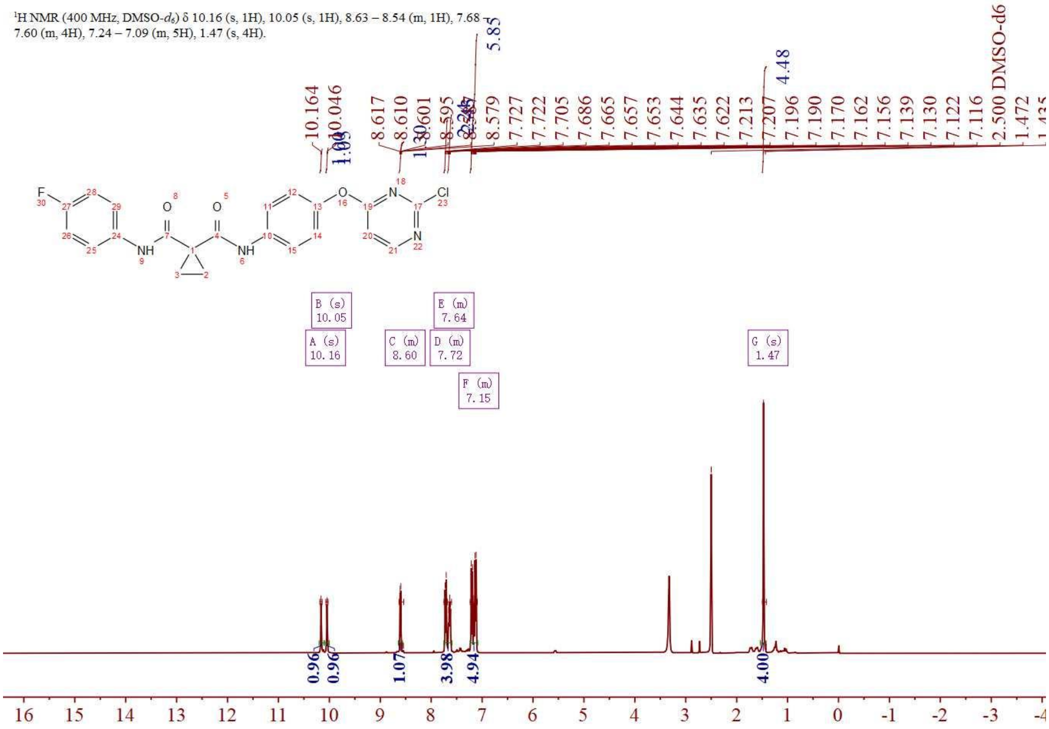

Figure S3. ¹H NMR of compound **3**

Spectra (^{1}H NMR, ^{13}C NMR, and LCMS) of molecule 10

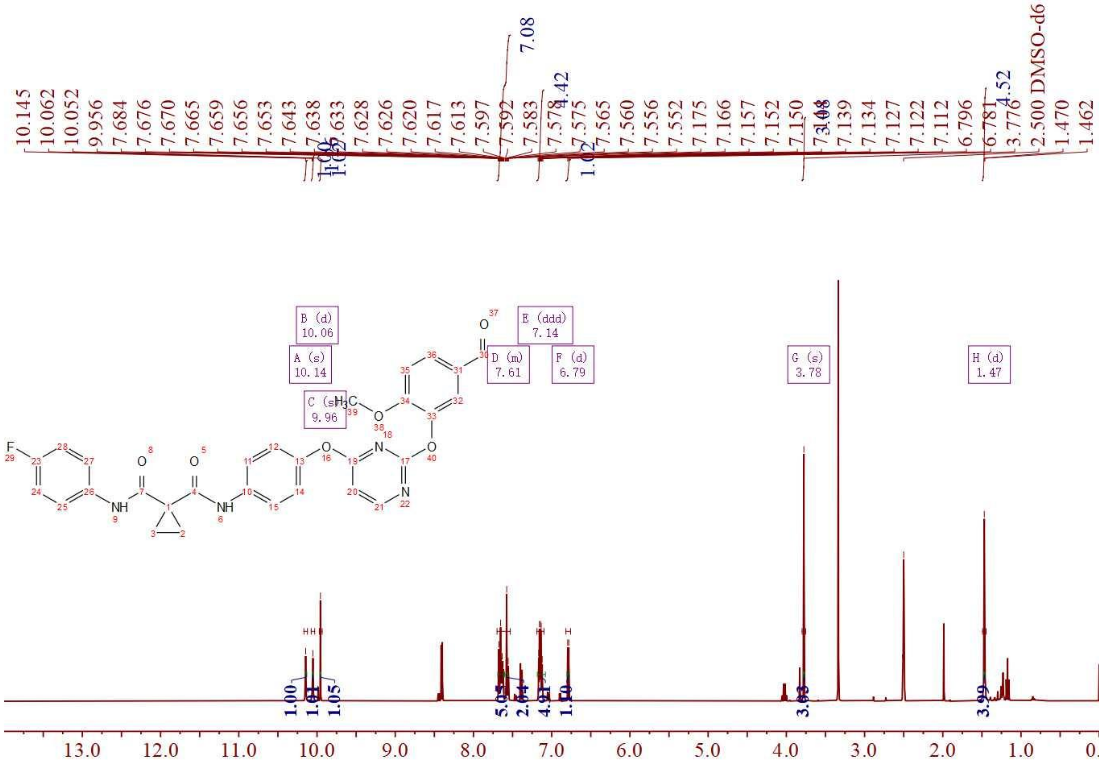

Figure S4. ^{1}H NMR of molecule **10**

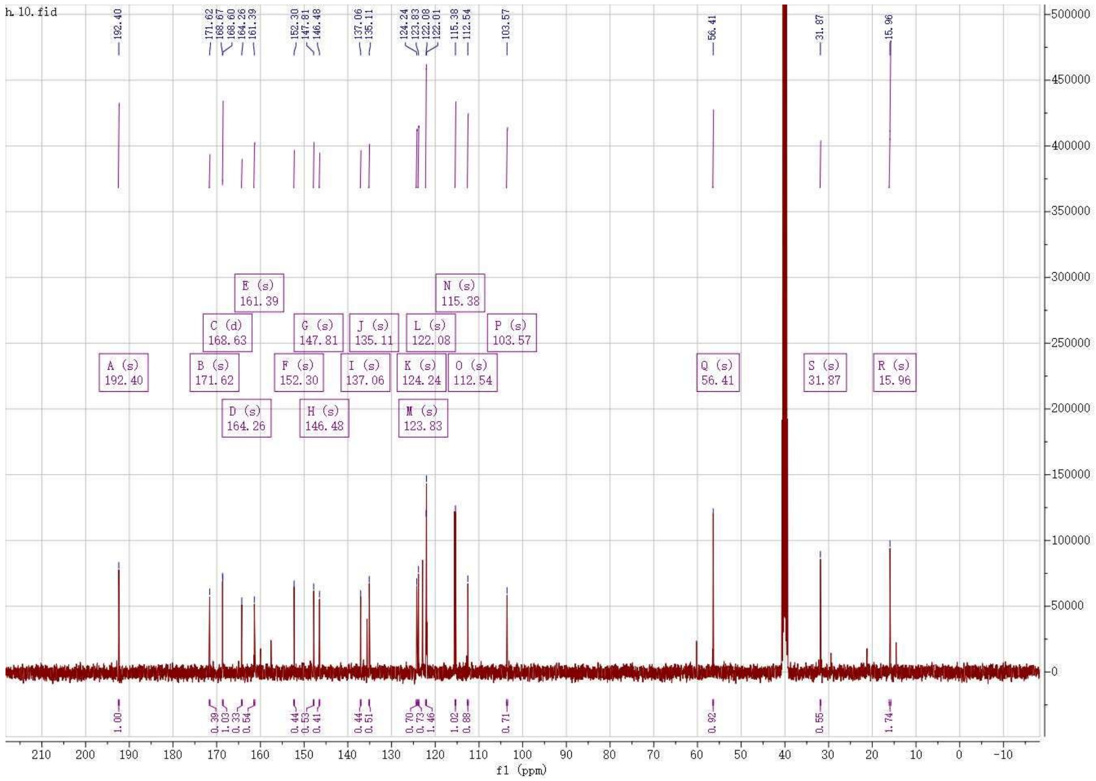

Figure S5. ^{13}C NMR of molecule **10**

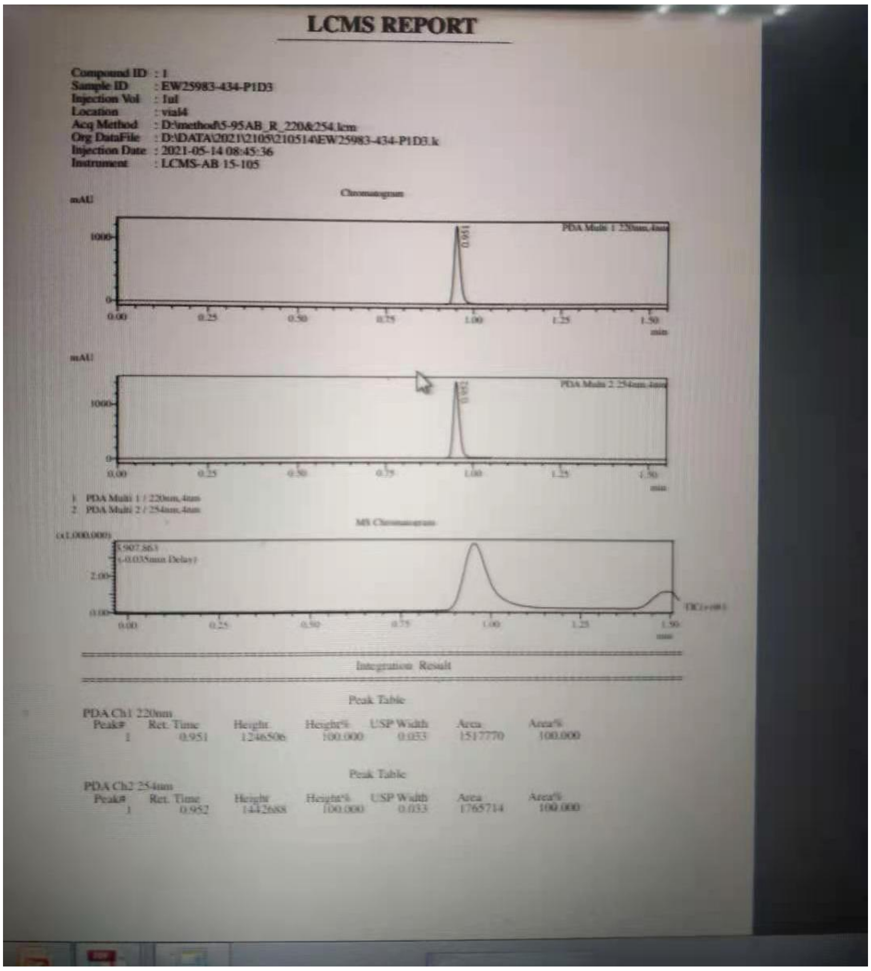

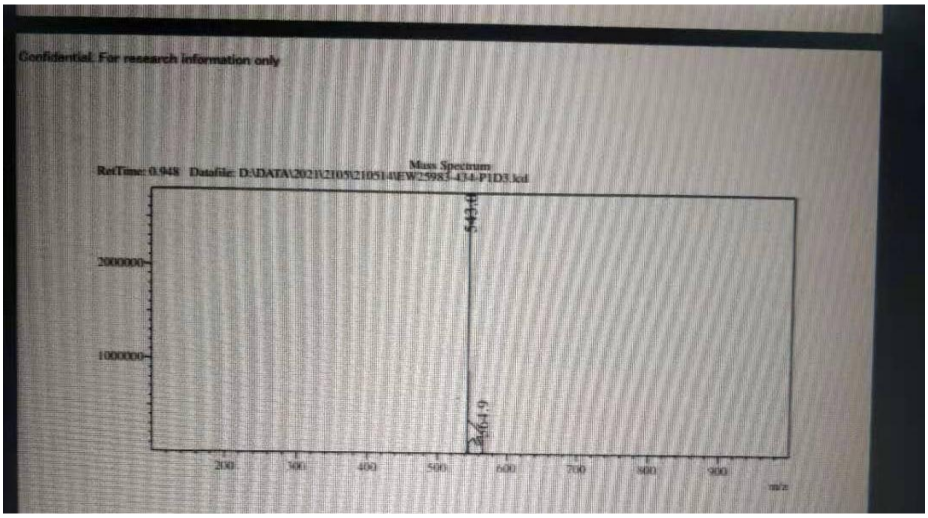

Figure S6. LCMS of molecule 10

3.1 Vitro anti-tumor activity experiment

MCF-7 Cells were added to DMEM high sugar medium (Hyclone) with 10% fetal bovine serum solution (Zhejiang Tian hang Biotechnology Co., Ltd.), 100 μmol/ml penicillin (Hyclone) and 100 mg/ml

streptomycin (Hyclone), and the cells were incubated at 37 ℃ in a 5% CO2 The cells were incubated in a CO2 incubator (Beijing Jing xi meng Instruments Co., Ltd.) at 37 ℃ and the culture medium was changed once a day, and the cells were ready for downstream experiments after the logarithmic growth phase.

Configuration of different concentrations of drugs: Weigh precisely 0.050 g of drugs to be tested numbered 10#, dissolve them in sterile DMSO and prepare a solution of 50 mg/ml, filter the solution through a 0.22 μm microporous membrane and store it at 4 ℃, this is the master batch of drugs to be tested. The working solutions were prepared at concentrations of 100 μg/ml, 10 μg/ml, 5 μg/ml and 2.5 μg/ml, respectively. Pentafluorouracil (5-FU) (Sigma) was used as the control and working solutions were prepared at concentrations of 100 μg/ml, 10 μg/ml, 5 μg/ml and 2.5 μg/ml.

MCF-7 Cells were obtained at logarithmic growth stage; the cell density was adjusted to 9×10^{4} cells/ml, gently mixed and inoculated in 96-well plates, ensuring 100 μl per well, and 150 μl of PBS buffer solution was added to the marginal wells for moisturization. 100 μl of different compounds (10#) was added to each well at final concentrations of 2.5, 5, 10 μg/ml. Normal control wells (normal incubation in DMEM medium) and positive control wells (incubation with concentrations of 10 μg/ml and 2.5 μg/ml pentafluorouracil, respectively) were set up at the same time, and three replicate experimental wells were set up for each concentration. After 24 h incubation in the incubator, the original culture solution was aspirated from the wells and each well was rinsed with 100 μl of PBS buffer solution. Each well was incubated for approximately 4 h in a CO2 incubator with 100 μl of MTT solution at a mass concentration of 1 mg/ml. The MTT solution was discarded and 150 μl of sterile DMSO solution was added to each well and gently shaken for 10 mins. The absorbance (A) value was then measured at a wavelength of 490 nm using an enzyme marker and the calculation of cell viability was completed using the following formula: 100% x (absorbance of experimental wells with drug added - absorbance of blank control wells without any drug added) / (absorbance of normal control wells - absorbance of blank control wells without any drug added) (absorbance of normal control wells - absorbance of blank control wells without any drug added).

We conducted an MTT assay to evaluate the biological activity of compound 10, using 5-fluorouracil (5-Fu) as a positive control. The experimental results demonstrated that compound 10 exhibits significant inhibitory effects on breast cancer cells, as shown in Figure S7. The IC50 value of compound 10 was determined to be 16.5 μg/mL, indicating a potent inhibitory activity at relatively low concentrations. These findings suggest that compound 10 possesses promising anti-breast cancer properties, providing a basis for further pharmacological studies and development.

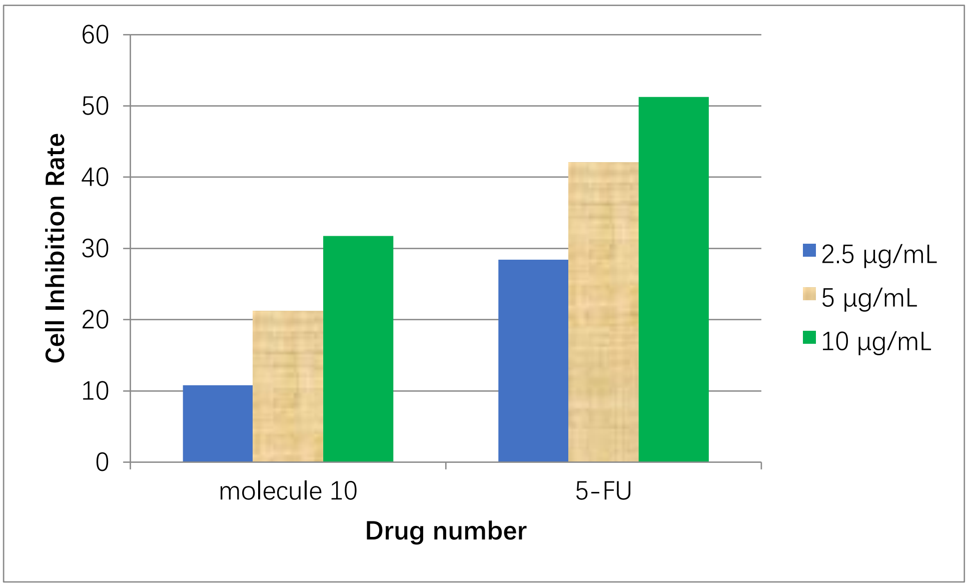

Figure S7 Calculate the cell inhibition rate of molecule 10 and 5–FU based on the measured OD value

Note:

Table 9 MCF-7 Cell Viability and IC50 Analysis

Compounds	IC_{50} (μM)
Molecule 10	0.32±0.026
5-FU（Positive cotrol）	0.45±0.012

Note:

IC50 values represent the mean ± standard deviation from three independent experiments.

The data were presented as the average of three replicates, with each experiment conducted in triplicate.

All values are expressed in μM.

Statistical significance was determined using a Student's t-test, with *P < 0.05 considered significant.
